# Loss of Cisd2 Exacerbates the Progression of Age-Related Hearing Loss

**DOI:** 10.14336/AD.2024.1036

**Published:** 2025-08-24

**Authors:** Hang-Kang Chen, Yen-Hsin Wang, Cing-Syuan Lei, Yu-Ru Guo, Ming-Chi Tang, Ting-Fen Tsai, Yi-Fan Chen, Chih-Hung Wang

**Affiliations:** ^1^Graduate Institute of Medical Sciences, National Defense Medical Center, Taipei 114201, Taiwan.; ^2^Department of Otolaryngology-Head and Neck Surgery, Tri-Service General Hospital, National Defense Medical Center, Taipei 114202, Taiwan.; ^3^The Ph.D. Program for Translational Medicine, Taipei Medical University, Taipei 11031, Taiwan.; ^4^Ph.D. Program for Cancer Molecular Biology and Drug Discovery, College of Medical Science and Technology, Taipei Medical University and Academia Sinica, Taipei 11031, Taiwan.; ^5^Department of Life Sciences and Institute of Genome Sciences, National Yang-Ming University, Taipei 11221, Taiwan.; ^6^Brain Research Center, National Yang-Ming University, Taipei 11221, Taiwan.; ^7^Aging and Health Research Center, National Yang-Ming University, Taipei 11221, Taiwan.; ^8^Genome Research Center, National Yang-Ming University, Taipei 11221, Taiwan.; ^9^Institute of Molecular and Genomic Medicine, National Health Research Institutes, Zhunan 35053, Taiwan.; ^10^Graduate Institute of Translational Medicine, College of Medical Science and Technology, Taipei Medical University, Taipei 11031, Taiwan.; ^11^Master Program in Clinical Genomics and Proteomics, School of Pharmacy, Taipei Medical University, Taipei 11031, Taiwan.; ^12^International Ph.D. Program for Translational Science, College of Medical Science and Technology, Taipei Medical University, Taipei 11031, Taiwan.; ^13^Graduate Institute of Microbiology and Immunology, National Defense Medical Center, Taipei 114201, Taiwan.

**Keywords:** Cochlea, Cisd2, Age-related hearing loss (ARHL), hearing impairment, hair cells, mitochondrial dysfunction

## Abstract

Age-related hearing loss (ARHL) is a disease that impacts human quality of life and contributes to the progression of other neuronal problems. Various stressors induce an increase in free radicals, destroy mitochondria to further contribute to cellular malfunction, and compromise cell viability, ultimately leading to functional decline. Cisd2, a master gene for Marfan syndrome, plays an essential role in maintaining mitochondrial integrity and functions. As shown by our data, specific deletion of Cisd2 in the cochlea exacerbated the hearing impairment of ARHL in C57BL/6 mice. Increased defects in mitochondrial function, potassium homeostasis and synapse activity were observed in the Cisd2-deleted mouse models. These mechanistic phenotypes combined with oxidative stress contribute to cell death in the whole cochlea. Human patients with obviously deteriorated ARHL had low Cisd2 expression; therefore, Cisd2 may be a potential target for designing therapeutic methods to attenuate the disease progression of ARHL.

## INTRODUCTION

Age-related hearing loss (ARHL; Presbycusis) is the third most common disability among elderly populations worldwide. In these populations, hearing impairment and loss may be detrimental to one’s emotional and mental health and could lead to social isolation as well as depression and even frustration. The process of ARHL is affected by many genetic or environmental factors, and there is still uncertainty about the mechanisms involved in presbycusis [1]. Histological analysis of postmortem tissues indicates that the causes of ARHL can be divided into four subtypes, namely, sensory (loss of hair cells), neural (loss of spiral ganglion neurons), strial (atrophy of the stria vascularis), and mechanical (thickening and stiffening of the basilar membrane) [2, 3]. The defects of ion transportation in spiral ligament and stria vascularis damage the homeostasis or potassium, subsequence bring about sensorineural hearing loss; additionally, programmed cell death in spiral ganglion and stria vascularis, induced by genetic defects or extrinsic stress, accelerates the pathogenesis of ARHL [4, 5]. Progressive hearing impairment is determined through measurements of pure-tone average, auditory brainstem responses (ABR), and distortion product otoacoustic emissions (DPOAEs); therefore, the elevation of auditory thresholds is related to base-to-apical degeneration of the cochlear hair cells and spiral ganglion neurons (SGNs). Some reports have shown that aging-induced SGN loss occurs as a consequence of hair cell loss, synapse loss, or both [6, 7]; some evidence has also suggested that cell loss in the spiral ligament is a major aspect of age-related cochlear degeneration, especially among the type IV fibrocytes [8]. In recent years, cumulative mitochondrial dysfunction has been believed to promote cellular senescence and further systemic aging and lead to the accumulation of ROS, which contributes to cell death. Therefore, loss of mitochondrial function is also a hallmark of ARHL.

Snapin, identified as a neuronal SNARE-binding protein, is enriched in synaptosomes and related to synaptic vesicles and late endosomes [9, 10]. Snapin could facilitate the synchronized fusion of synaptic vesicles in neurons and participate in the late endosomal transport of the autophagic process [9, 11, 12]. To regulate synaptic activity, Snapin coordinates endolysosomal sorting and trafficking functions to control pool size and neurotransmitter release [12]. The important functions of mitochondria in neurons, in addition to ATP production, are the regulation of redox signaling and calcium homeostasis [13]; otherwise, mitochondria participate in regulating the processes of synaptic plasticity and functions.

The CISD2 protein, translated from the CISD2 gene (also named Noxp70, MINER1, ZCD2, ERIS and Naf-1), is the second member of the protein family containing the CDGSH iron sulfur domain. The CISD2 gene is located within the region on human chromosome 4q22-24 where a genetic component for human longevity has been mapped through a comparative genome analysis of centenarian siblings [14]. The expression levels of Cisd2 decline with age, and Cisd2 knockout mice have defects in nerve and muscle systems around the weaning stage and develop additional premature aging phenotypes [15-17]. As previous data have shown, Cisd2 is localized to mitochondria and the endoplasmic reticulum (ER), which are the main intracellular calcium stores for modulating calcium homeostasis. Cisd2 interacts with Bcl-2 and forms a complex with the inositol trisphosphate receptor (IP3R) to regulate calcium release [18]. Cisd2 also interacts with Gimap5 on mitochondria-associated ER membranes (MAMs) to regulate the mitochondrial calcium buffering capacity [19]. Previous studies have identified the CISD2 gene as causative for WFS2, and a G-to-C transversion in nucleotide 109 in exon 2 of the *CISD2* gene was identified in 3 consanguineous families of Jordanians with WFS2 [20]. Individuals with WFS present juvenile-onset insulin-dependent diabetes mellitus and optic atrophy [21] with neurological and endocrine manifestations including diabetes insipidus, sensorineural deafness, dementia, psychiatric illnesses, renal-tract abnormalities and bladder atony [22, 23]. Additionally, mitochondrial dysfunction is not only observed in the aging process but also contributes to the pathogenesis of diseases, such as neurodegenerative diseases and hearing loss. Although high glycolytic (anaerobic) metabolism was observed in outer hair cells, quantitative analysis of the contributions of glycolysis and oxidative phosphorylation has clearly established that their metabolism is primarily aerobic [24]. Outer hair cells are more susceptible to oxidative stress due to their lower antioxidant content [25]. Evidence from human and animal studies indicated that the mitochondrial metabolic effects on ARHL must be more complex than simply generating excess reactive oxygen species [26].

There are many possible causes of ARHL, such as heredity, disease and environmental factors. However, it is still not clear which gene(s) and signaling pathways of the inner ear play a critical role in hearing loss during the aging process. There are many genes involved in the pathogenesis of ARHL, deafness-causing genes, neurotransmitter-related genes, oxidative stress-related genes and mitochondrial function-related genes. Loss of Cisd2 led to mitochondrial dysfunction and increased oxidative stress. Therefore, we aimed to investigate the role of the age-related gene Cisd2 in ARHL and decipher the molecular mechanism involved in ARHL using tissue-specific gene knockout mouse models.

## MATERIALS AND METHODS

### Study approval

Our study enrolled 21 participants, including 8 patients with ARHL (mean age: 58.7 years old) and 13 subjects with normal hearing (mean age: 27.6 years old). We excluded people with a history of ear trauma, previous ear surgery, and otitis media. Before performing the hearing tests, all participants underwent otoscopic examinations by an otologist to ensure the patency of the external auditory canal and to exclude any ontological diseases. The Tri-Service General Hospital Institutional Review Board approved the research project (TSGHIRB 1-103-05-112). All of the mice in this study were bred and housed in a specific pathogen-free (SPF) facility; the animal protocol was approved by the Institutional Animal Care and Use Committee (IACUC) of the National Defense Medical Center Laboratory Animal Center (approval numbers: IACUC 15-039).

### Cisd2 knockout mouse models

Conventional Cisd2 knockout (Cisd2 conKO) and Cisd2 floxed allele (Cisd2^flx/flx^) mice were generated as previously described [15, 19]. The Nestin-Cre transgenic (Nes-Cre, JAX no. 003771) mouse model was purchased from the Jackson Laboratory and bred with Cisd2^flx/flx^ mice. The constructed design and model generation of the Otoa-Cre transgenic mouse model was constructed and generated by the National Laboratory Animal Center, Taiwan. The genotypes of neuron-specific knockout (Rrm2b neKO; Nes-Cre/+; Cisd2^flx/flx^) and cochlea-specific knockout (Rrm2b coKO; Otoa-Cre/+; Cisd2^flx/flx^) mouse models were detected using regular PCR analysis (Supplementary Table 1). In this study, all we used were male mice. Gt(ROSA)26Sor^tm4(ACTBtdTomato,EGFP)Luo^/J (ROSA^mT/mG^, stock number: 003474) mice were purchased from The Jackson Laboratory. Originally, all the cells show orange fluorescence (tdTomato) in this mouse model. If the cells express Cre recombinase, they exhibit green fluorescence (eGFP).

### Auditory brainstem response (ABR) and distortion product otoacoustic emissions (DPOAE)

Mouse auditory function was assessed by evoked ABR. In brief, mice were anesthetized with an intraperitoneal injection of xylazine (16 mg/kg) and ketamine (100 mg/kg) and kept warm with a heating pad in a sound-attenuating chamber. Subdermal needle electrodes were inserted at the vertex (positive), below the pinna of the ear (negative), and at the back (ground) of the mice. Specific stimuli (clicks and 4-, 8-, 16-, and 32-kHz tone bursts) were generated using SigGen software (Tucker-Davis Technologies) and delivered to the external auditory canal. The average responses from 1024 stimuli for each frequency were obtained by reducing the sound intensity in 5-dB steps until the threshold was reached. Thresholds were defined as the lowest intensity at which a reproducible deflection in the evoked response trace could be recognized [27].

The distortion-product otoacoustic emissions (DPOAEs) were measured at center frequencies (CFs) of 4, 8, 12, 16, 24, 28 and 32 kHz with a real-time signal processing system (Tucker-Davis Technologies, Gainesville, FL, United States), as described previously [28]. Two simultaneously presented pure tones, F1 and F2, were calculated using the CF, where F1 was CF × 0.909 and F2 was CF × 1.09. This yielded a frequency of primary 1 (Tone 1) and primary 2 (Tone 2) geometrically centered on the CF. The two primary tones were presented at the same intensity (L1 = L2 = 65 dB) and at a frequency ratio (F2: F1) of 1:2. The primary tones produced by two separate speakers (EC1 close-field speakers; Tucker-Davis Technologies) were transmitted into each animal’s ear canal. DPOAE recordings were made with a low-noise microphone (ER 10B+; Etymotic Research, Elk Grove Village, IL, United States) and averaged 512 times at each frequency. The peak of the cubic difference distortion product (2F1-F2) at various CFs was accepted as a DPOAE if it was 3 dB above the noise floor. The difference was referred to as the signal-to-noise ratio (SNR).

### Human sample preparation and quantitative real-time PCR

5 mL of equine blood was collected into an EDTA blood collection tube, centrifuged at 2000 × g at 4°C for 10 min. Plasma was removed and WBC were transferred into a 7.5 mL tube. 3-times the volume of RBC lysis buffer (79217, Qiagen) was added. The tube was vortexed for 15 sec and incubated at room temperature for 15 min prior to centrifugation at 2000 × g for 10 min (4°C). Supernatant was discarded, the isolation of RNA from WBC pellet was performed using TRIzol® reagent (15596-026, Invitrogen Life Technologies) following the manufacturer’s instructions. For concentration, purity and quality control of the isolated RNA we used a Nanodrop (Nanodrop Instruments). Reverse transcription of 450 ng RNA was done with the Roche Transcriptor First Strand cDNA Synthesis Kit (04896866001, Mannheim). A quantitative polymerase chain reaction (qPCR) was performed using the Applied Biosystems TaqMan CISD2 (Hs 00381903) and GAPDH (Mm 99999915) probe assay. Targeted for CISD2 and GAPDH was performed in a reaction made up of 3.5 µL nuclease-free water, 1 µL primer/probe, and 10 µL TaqMan universal PCR Master Mix (4318157, Thermo Fisher Scientific) combined with 1 µL cDNA. This qPCR reaction was performed on the Applied Biosystems 7500 Fast Real-Time PCR instrument. Instrument settings included a fast mode step, with pre-PCR heating at 60 °C. A polymerase activation step for 10 minutes at 95 °C, denaturation for 15 seconds at 95 °C, and an anneal/extension step for 1 minute at 60 °C was performed, for a total run time of 40 minutes.

### Mouse RNA extraction and quantitative real-time PCR

Total RNA was isolated from cochlea and brain tissues of mice using TRIzol Reagent (15596-026, Invitrogen Life Technologies). We executed real-time quantitative PCR using a TaqMan probe with TaqMan^®^ Fast Universal PCR Master Mix and a real-time PCR instrument (Thermo-Fisher Scientific). All amplifications were performed in triplicate for each RNA sample and primer set. The amount of total input cDNA was calculated using hypoxanthine-guanine phosphoribosyltransferase (Hprt) as an internal control (Supplementary Table 2).

### Histological analysis

The cochleae were dissected and perfused with 4% paraformaldehyde (in 0.1 M phosphate buffered saline [PBS], pH 7.4) through the opened oval window and a small hole in the apex. After a 2 h post-fixation, the cochleae were decalcified in 10% ethylenediaminetetra-acetic acid (EDTA), pH 7.3, at 4°C on a rotating shaker; the EDTA solution was changed daily until decalcification was complete. After immersion in a graded sucrose series (15, 20, and 25%) for 30 min, and overnight immersion in 30% sucrose at 4°C, the cochleae were embedded in paraffin, sectioned at 5 μm, and immunostained using the Mouse/Rabbit PolyDetector HRP/DAB Detection System (BSB 0261, Bio SB Inc.) and standard procedures for quenching (H_2_O_2_ in methanol) and blocking (PolyDetector Peroxidase Blocker) endogenous peroxidase activity and for blocking non-specific antibody binding (1xPBS containing 3% horse serum and 0.3% Triton™ X-100). The slides were blotted, covered with antibody dilution buffer (S3022, Dako Co.) containing primary antibody (for target protein), and incubated in a humidified chamber for 2 h at RT, washed, and incubated with PolyDetector Label HRP secondary antibody for 30 min at RT. The slides were washed again, treated with PolyDetector DAB substrate-chromogen solution for 10 min, rinsed in distilled water, counterstained with hematoxylin (Muto Pure Chemicals Co., Ltd.), dehydrated through a graded alcohol series (50-100%), cleared in xylene, and mounted in Fluoromount-G mounting medium (010-20, Southern Biotech). The slides were examined using an Olympus BX50 microscope equipped with a digital camera (Olympus DP74, Olympus Corp.).

The brain was collected, fixed with formalin and embedded in paraffin. Serial sections from each sample were subjected to staining analysis. We performed hematoxylin & eosin staining (H&E) to observe the histology of tissues with or without Cisd2 expression. We also carried out immunohistochemistry (IHC) staining to monitor the expression of specific proteins. The paraffin-embedded tissue sections were incubated with antibody and detected with the LSABTM Kit (DakoCytomation) according to the manufacturer’s instructions.

The antibodies we used were LC3B antibody (2775S, Cell Signaling), SNAPIN (GTX64946, GeneTex), Cisd2 (NBP1-84809, Novus Biologicals) and CISD2 (PAB20855, Abnova) for IHC staining and Mayer Hematoxylin and Eosiin-Y Sol. (Histo-Line Laboratories) for H&E staining.

### Confocal microscopy

The tissues were then incubated with anti-carboxyl-terminal binding protein 2 (CtBP2) IgG1 (612044, BD Biosciences, San Jose, CA, USA) for 2 h. After three washes with PBS, the tissues were incubated with Alexa Fluor 488-conjugated goat anti-mouse IgG1 (γ1) (A21142, ThermoFisher Scientific, Waltham, MA, USA) antibodies for 1 h. The samples were incubated with Alexa Fluor 633-conjugated phalloidin (A22284, Thermo Fisher Scientific, Waltham, MA, USA) for 1 h at room temperature, rinsed with PBS, mounted with DAPI Fluoromount-G® mounting medium (0100-20, SouthernBiotech, Birmingham, AL, USA), and covered with a coverslip for analysis. Fluorescence images were obtained using a confocal laser scanning microscope (Zeiss LSM 880, Carl Zeiss, Jena, Germany).

### Ultrastructure observation

Mouse auditory nerves were fixed in a mixture of glutaraldehyde (1.5%) and paraformaldehyde (1.5%) in cacodylate buffer, postfixed in 1% OsO4, and then rinsed in cacodylate buffer. Following dehydration, the tissues were embedded and sectioned for TEM as described previously [29]. The ultrastructure was observed using a transmission electron microscope (Hitachi HT-7700).

### Protein extraction and Western blotting

Mouse tissue samples were homogenized in lysis buffer (50 mM Tris at pH 7.4, 100 mM NaCl, 1 mM EDTA, 1% Triton X-100 with complete protease inhibitor and phosphatase inhibitor cocktails [Roche]) and denatured in sample buffer (50 mM Tris at pH 6.8, 100 mM Dithiothreitol, 2% SDS and 10% glycerol). The extracted proteins were separated on SDS-PAGE gels and electrotransferred onto polyvinylidene difluoride (PVDF) membranes (GE Healthcare). After blocking with 5% (w/v) nonfat dry milk, the membrane was hybridized with the indicated primary antibodies and corresponding HRP-conjugated secondary antibody and finally detected using a Visualizer Kit (Millipore WBKLS0500). All the data were normalized against an internal control, actin (1:1000, MAB1501, Millipore), β-actin (ab10983, abcam) or glyceraldehyde 3-phosphate dehydrogenase (GTX 100118, Gapdh). The primary antibodies used in this study were from the following sources: polyclonal antibody against Cisd2 (1:1000, homemade) [15].

### Coimmunoprecipitation assay (IP)

After Cisd2 knockdown HEI-OC1 cells were washed, and 500 µl RIPA buffer was added to the cell pellet and centrifuged for 15 min at 4°C. The supernatant was transferred to a new tube, and 4 µg of antibody (SNAPIN, GTX64946, GeneTex) was added and shaken for 2~3 hr at 4°C. Fifty microliters of protein A/G was added, and the mixture was shaken for 1 hr at 4°C. After 1 hr, the samples were centrifuged at 8000 rpm for 1 min at 4°C, and the supernatant was removed. The samples were then washed in RIPA wash once and twice in 1X PBS. Then, 50 µl of 2X sample buffer was added, centrifuged at 8000 rpm for 1 min at 4°C and subjected to SDS-PAGE. Western blotting was performed with both the SNAPIN (GTX64946, GeneTex) and CISD2 (PAB20855, Abnova) antibodies.

### Measurement of mitochondrial bioenergetic functions

The oxygen consumption rate (OCR) of mitochondria was detected by an XFe24 analyzer (Seahorse Bioscience, MA) [19]. Primary cells were cultured in a 24-well microplate at 6 × 10^3^ cells/well, kept overnight in normal growth medium and switched to sodium bicarbonate-free William’s E medium (Sigma) supplemented with 2% FBS, 2 mM glutamine, 100 U/ml penicillin, 100 μg/ml streptomycin, 1 mM sodium pyruvate and 1% insulin-transferrin-selenium (ITS) at 1 hour before measurement. The OCR and ECAR were measured before and after adding the indicated chemicals (2 μM oligomycin A, 1 μM FCCP and 1 μM rotenone and antimycin A) at 37°C.

### Analysis of autophagy

Antigen accessibility was increased by treatment with 0.1% saponin for 10 min in BlockPRO blocking buffer (Visual Protein Biotechnology, Taipei, Taiwan). After PBST washes, nonspecific antibody binding was blocked by BlockPRO blocking buffer for 60 min at RT. The cells were incubated with polyclonal primary antibodies against LC3A/B (1:100; ab62721, Abcam) in an antibody dilution buffer (Dako Co.), incubated in a humidified chamber for 1 h at RT, and, after being washed with PBST, stained with a secondary antibody (donkey anti-rabbit Alexa Fluor 488, 1:500, Molecular Probes) for an additional 60 min. After being washed three times with PBST, the cells were mounted in DAPI Fluoromount-G (SouthernBiotech). Cell images were captured with an Olympus BX50 brightfield/fluorescence microscope (Olympus Corp.) equipped with a digital camera (Olympus DP74). Digital photomicrographs were processed with the cellSens Standard 1.17 (Olympus) software. This protocol was modified from previous reports [30].

### Terminal Deoxynucleotidyl transferase dUTP nick end labeling (TUNEL) assay

Paraffin-embedded cochlear sections were dewaxed in xylene and rehydrated, and TUNEL assays were performed according to the instructions provided with the *in situ* Cell Death Detection Kit, POD (Roche) as previously described [28]. Deparaffinized slides were incubated with 3% H_2_O_2_ in methanol to block endogenous peroxidase activity and permeabilized with 0.1% Triton X-100 in 0.1% sodium citrate. The tissues were blocked with blocking buffer (Tris-HCl, 0.1 M/3% BSA/20% normal bovine serum) and incubated with the TUNEL reaction mixture in a humidified atmosphere in the dark. After washing with PBS Tween-20, the tissues were stained with Converter-POD. Labeled apoptotic cells were identified by treating the slides with DAB substrate, and the nuclei were counterstained with hematoxylin. Slides were examined with an Olympus BX50 brightfield/fluorescence microscope (Olympus Corp.) equipped with a digital camera (Olympus DP74). Digital photomicrographs were processed with the cellSens Standard 1.17 (Olympus) software.

### ATP measurement

For the determination of cochlear lysate ATP levels, an ATP Bioluminescence Assay Kit CLS II (Roche Applied Science) was used following the manufacturer’s protocol. The lysate supernatant reacted with luciferase reagent, and the luminescence intensity was measured using a Multi-Mode Reader (Synergy 2, BioTek Instruments).

### Lactate determination

To measure the amount of lactate in the cochlea lysate, lactate content was measured using the Lactate Colorimetric Assay Kit II (BioVision) according to the manufacturer’s instructions. Lactate was oxidized using lactate dehydrogenase to generate a product that interacts with a probe to produce a color (max = 450 nm) measured using a Multi-Mode Reader (Synergy 2, BioTek Instruments).

### Cisd2 knockdown in the HEI-OC1 cell line

Small hairpin RNAs (shRNAs) designed for the Cisd2 gene (shCisd2) were introduced into the expression plasmid pLKO.1, which was purchased from the RNAi Core Facility at Academia Sinica, Taipei, Taiwan. The target sequences were as follows: shCisd2 #1 and #2, 5′-GCAACAGAAGGATA GCTA-3′ and 5′-CCCAAGG TGGTGAATGAGA-3′, respectively. pLKO.1-shCisd2 was transfected into the HEI-OC1 (House Ear Institute-Organ of Corti 1) cell line, and stable clones with pLKO.1-shCisd2 were selected by puromycin treatment.

### Statistical analysis

All the results are presented as the mean ± SD from four or more independent samples. Comparisons between two groups were calculated using a 2-tailed heteroscedastic Student’s t-test. Repeated measures ANOVA was applied for ABR and DPOAE analysis followed by Tukey’s HSD for multiple comparisons using SPSS (version 18). The statistical analysis for normal distribution was determined with t-test or ANOVA; for not normal distribution was determined with Mann-Whitney U test (two groups) or Kruskal-Wallis test (group number more than 2).

**Figure 1.**
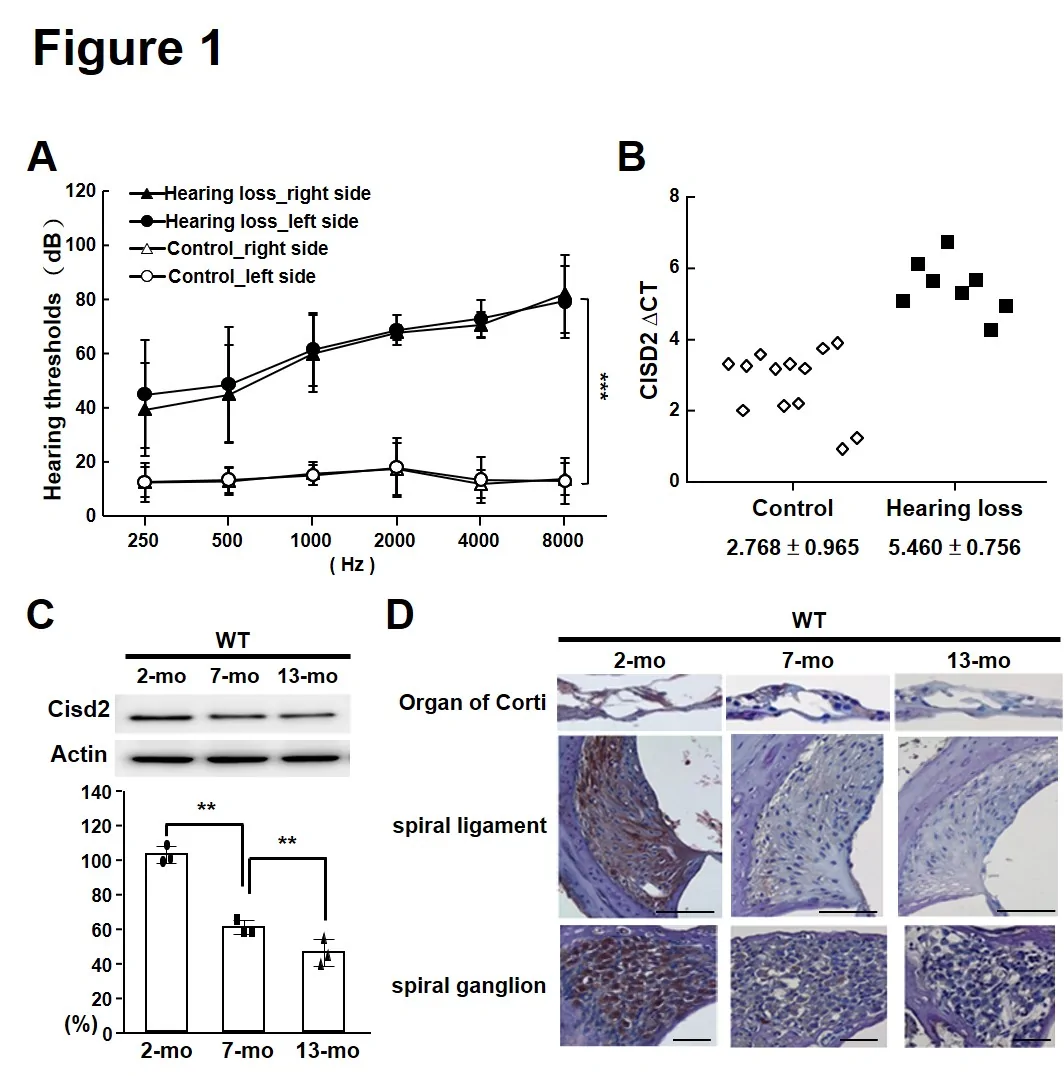
**A decrease in Cisd2 expression levels in hearing-loss and aged individuals**. (**A**) Higher hearing thresholds at different frequencies of both ears, mainly the high frequency, were observed in patients with ARHL. (**B**) Lower mRNA expression of Cisd2 was observed in the ARHL group (n=8) compared to the normal hearing one (n=13). (**C**) Age-dependent declines in Cisd2 protein expression levels were observed in the cochlear extracts of WT mice at 2, 7 and 13 months of age. N = 3 mice per group. (**D**) Immunohistochemical analysis of Cisd2 staining. In the organ of Corti, spiral ligament and spiral ganglion, the protein levels of Cisd2 decreased in an age-dependent manner according to immunohistochemistry staining. The results are presented as the mean ± SD. **p<0.01; ***p<0.001. Scale bar, 50 μm.

## RESULTS

### Low expression of Cisd2 in patients with hearing loss

*Cisd2* is a candidate gene for WFS2, and one critical impairment of WFS2 patients is hearing loss. In our previous studies, Cisd2 deficiency led to mitochondrial dysfunction resulting in neurodegeneration, which may induce hearing impairment. Samples of human patients with an accelerated ARHL were collected, and the expression levels of *CISD2* were detected (Fig. 1A). Decreased expression levels of *CISD2* were observed in these human patients compared to normal hearing subjects (Fig. 1B). The *Cisd2* expression levels were age-dependently decreased in the cochlea of wild-type (WT) mice, including the organ of Corti, spiral ligament and spiral ganglion (Fig. 1C-D). Therefore, Cisd2 may be involved in modulating hearing functions, and lower Cisd2 expression could lead to hearing impairment.

**Figure 2.**
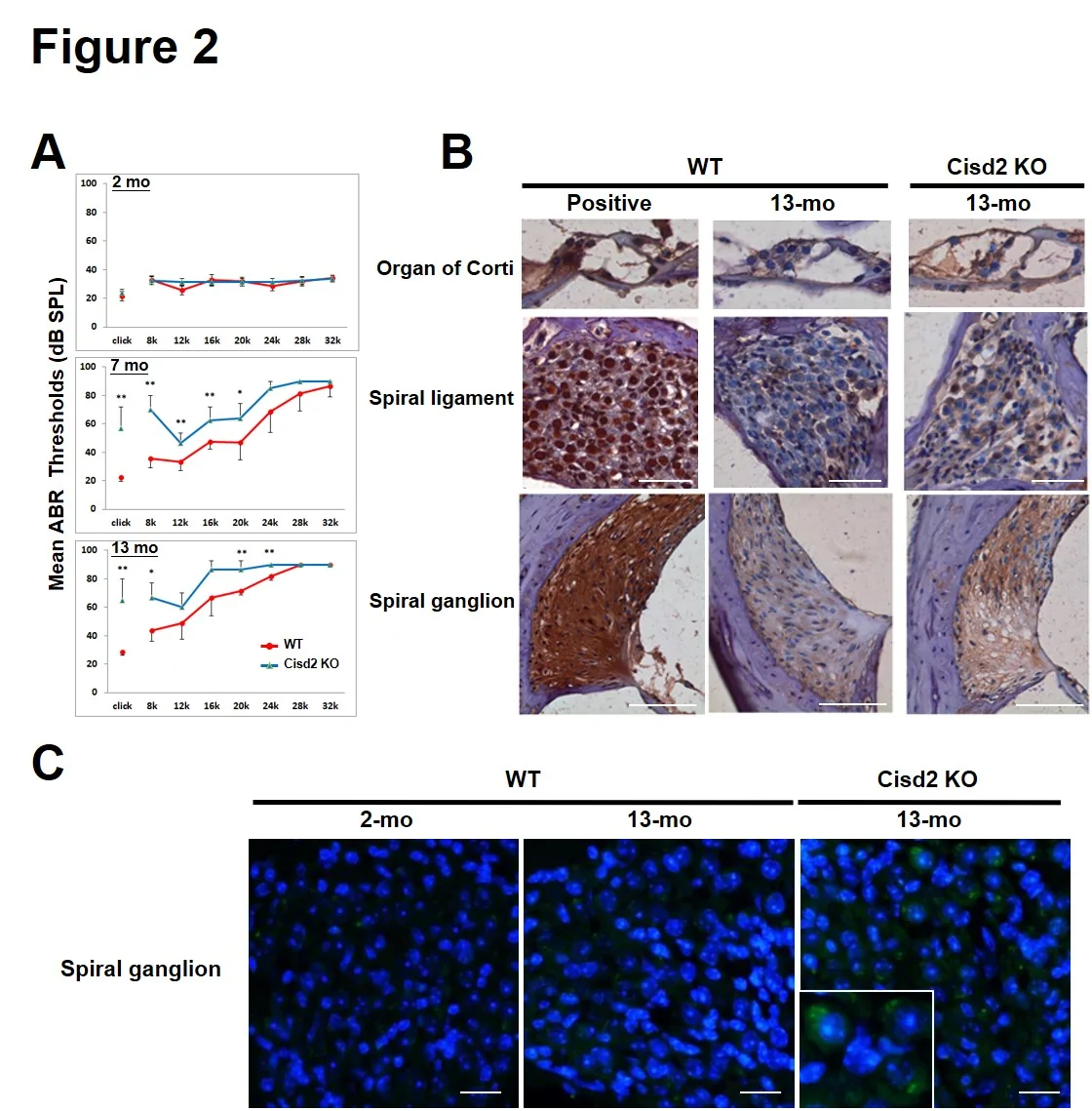
**Hearing loss and cellular loss of cochlea observed in the Cisd2 KO mice**. (**A**) No obvious difference in ABR in the WT and Cisd2 KO mice at 2 months of age. Hearing impairment was observed in the 7- and 13-month Cisd2 KO mice. N = 12 mice per group. The results are presented as the mean ± SD. *p<0.05; **p<0.005. (**B**) Analysis of apoptosis in the WT and Cisd2 KO mouse cochlea by TUNEL staining. Positive controls were tissue slides treated with 100 U/mL DNase I for 15 min. Scale bar, 50 μm. (**C**) Immunohistochemistry staining of LC3 in the spiral ganglion obtained from 2-mo and 13-mo WT and 13-mo Cisd2 KO mice. More LC3 puncta were detected in the ganglion cells of Cisd2 KO mice. The inside figure indicates the accumulated expression of LC3 in the cytoplasm. Scale bar, 25 μm.

### Hearing impairment in Cisd2 knockout mice at middle age

**Figure 3.**
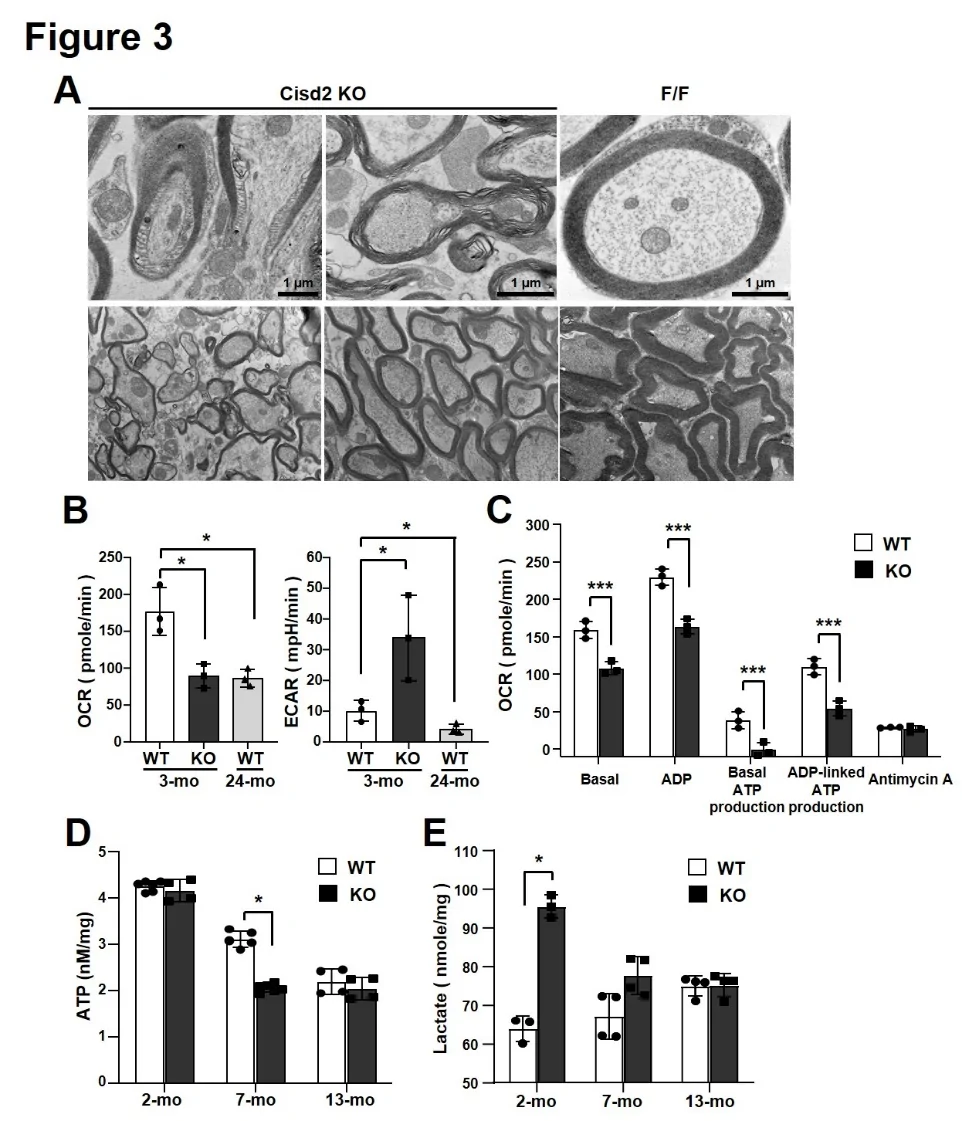
**The effects of Cisd2 deletion on mitochondrial functions**. (**A**) Auditory nerves in the cochlea were collected from the Cisd2 KO and F/F mice at 3 months old. Ultrastructural data from different parts of auditory nerves were obtained using transmission electron microscopy (TEM). (**B**) Cisd2 deletion led to lower mitochondrial function and higher glycolytic function in the cochlea. Aged mice had lower mitochondrial function and lower glycolytic function in the cochlea. N=3. (**C**) Effect of Cisd2 expression on coupling in mitochondria isolated from mouse cochleae. Mouse age, 2 months old. N=3. (**D**) ATP generation was obviously not different between Cisd2 KO and WT mice at 2 months of age. ATP generation in WT cochleae was decreased by 25% at 7 months of age and by 50% at 13 months of age. ATP generation in the Cisd2 KO cochlea was decreased by 50% at 7 and 13 months of age. (WT mice at 2, 7 and 13 months old, n= 6, 5 and 4 respectively. Cisd2 KO mice at 2, 7 and 13 months old, n= 4, 6 and 4 respectively). (**E**) Lactate generation of the Cisd2 KO cochlea was higher than that of the WT cochlea at 2 months old but was not obviously different at 7 and 13 months old. (WT mice at 2, 7 and 13 months old, n= 3, 4 and 4 respectively. Cisd2 KO mice at 2, 7 and 13 months old, n= 3, 4 and 4 respectively). The results are presented as the mean ± SD. *p<0.05; **p<0.01; ***p<0.001.

C57BL/6 mice present the classic features of ARHL by 12-15 months of age [31]. Several morphological alterations are found in C57BL/6 mice, including loss of hair cells, defects of spiral ganglion cells and atrophy of the stria vascularis [32]. Previous reports have also shown that WFS patients with Cisd2 deletion have severe hearing impairment and even hearing loss. As our data revealed, the Cisd2 KO mice had high ABR thresholds at 7 months and 13 months old but no significant defects in hearing at 2 months old compared to WT mice (Fig. 2A). The Cisd2 KO mice showed defects and decreased cell numbers in the organ of Corti and spiral ligament (Supplementary Fig. 1A-C) and alterations in cellular morphology in the spiral ganglion (Supplemental Fig. 1D-E). In addition, the Cisd2 KO mice had more apoptotic cell death in the cochlea, such as the positions of the organ of Corti, spiral ligament and spiral ganglion, at 13 months of age, as determined using the TUNEL assay (Fig. 2B; Supplementar Fig. 1F). The autophagosome marker LC3 was more highly expressed in the spiral ganglion cells of Cisd2 KO mice (Figure 2C). Neuronal degeneration with age also contributes to hearing impairment in elderly individuals. Smaller and impaired axons of auditory nerves in the cochlea were observed in the Cisd2 KO mice (Fig. 3A). Measurement of energy metabolism revealed lower oxidation, especially at basal, state 3 and state 3μ, and higher glycolysis in the cochlea of Cisd2 KO mice compared to WT mice (Fig. 3B-C); moreover, decreased ATP and increased lactate generation were observed in Cisd2 KO mice (Fig. 3D-E).

The aging effect also diminished the oxygen consumption rate (OCR) and extracellular acidification rate (ECAR) in WT mice. Young Cisd2 KO mice had low OCR as aged WT mice shown; interestingly, they had dramatically higher ECAR than WT mice (Fig. 3B). These data indicated that there were more dysfunctional organelles and impaired cells in the cochlea of Cisd2 KO mice, and consequently, the induction of autophagy and apoptosis contributes to hearing impairment and even hearing loss. Accordingly, loss of Cisd2 could exacerbate and accelerate the phenotypes of ARHL in C57BL/6 mice.

**Figure 4.**
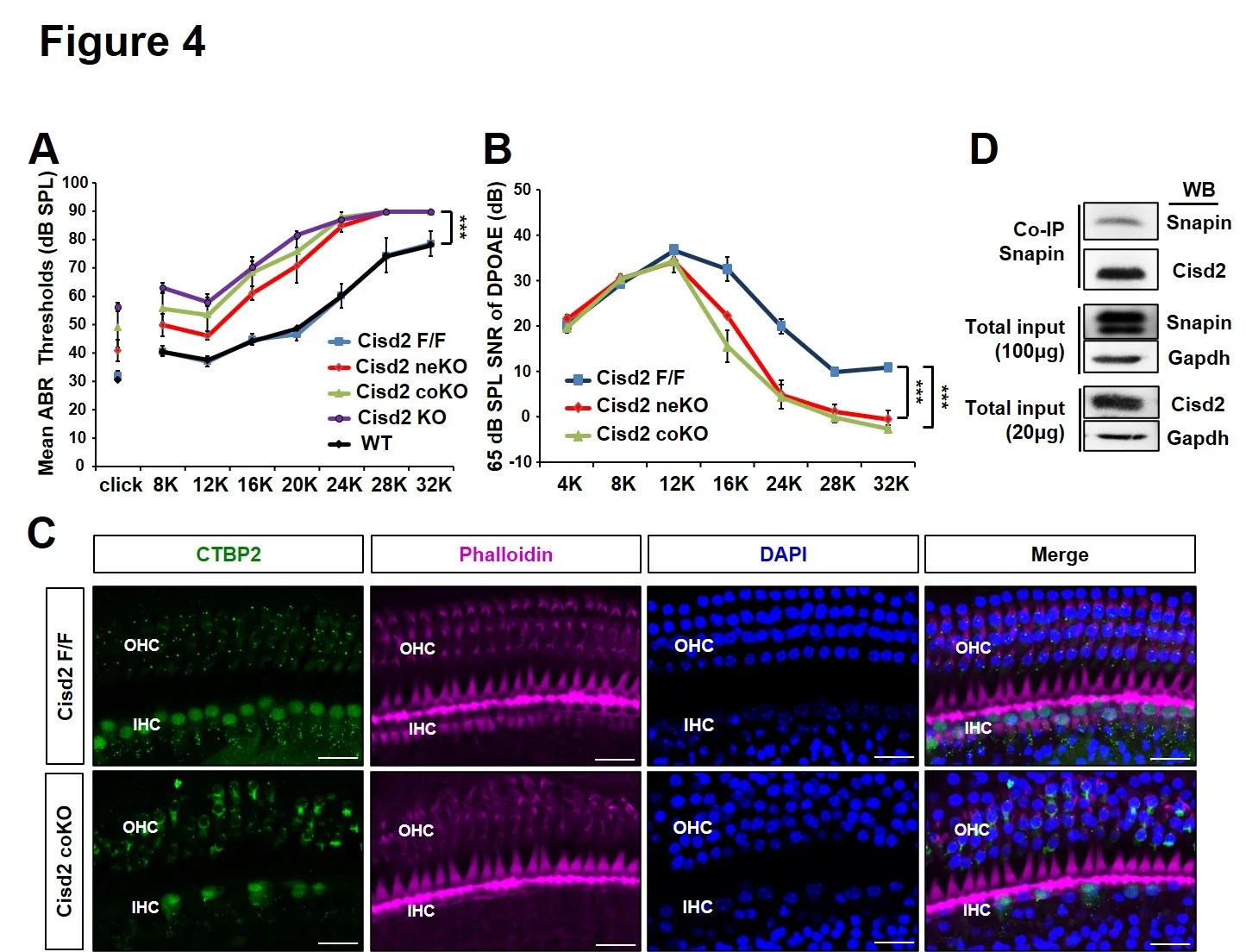
**Synaptic defects lead to impaired healing function in Cisd2 knockout mouse models**. (**A**) Hearing impairment (under all conditions) was observed in the Cisd2 conventional KO (conKO) and two conditional KO (coKO and neKO) mice. Mouse age, 6-8 months old. (**B**) Loss of the DPOAE response was observed in conditional KO (coKO and neKO) mice. Mouse age, 6-8 months old. (**C**) CtBP2 (green) was significantly decreased in the Cisd2 coKO mice at 3 months old. DAPI (blue) was used to stain nuclei, CtBP2 (green) was used to stain synaptic ribbons, and phalloidin (pink) was used to stain actin filaments (F-actin). Scale bar, 20 μm. (**D**) The interaction between Snapin and Cisd2 was analyzed using co-IP and Western blot analysis. N = 6 mice per group. The results are presented as the mean ± SD. ***p<0.001.

### Hearing impairment in Cisd2 tissue-specific KO mice

Defects in the cochlea and neurons could lead to hearing loss, especially ARHL. We generated mice with cochlea-specific expression of Cre recombinase, Otoancorin (Otoa)-driven Cre recombinase (Supplementary Fig. 2A). Cre recombinase could be expressed in the cochlea, as our data showed (Supplementary Fig. 2B). Nestin-driven Cre recombinase could be expressed in the whole brain (most subregions) and spinal cord (Supplementary Fig. 2C-D). The Cisd2 tissue-specific (cochlea and neuron) knockout mouse models were generated using our breeding strategy and genotyping (Supplemental Fig. 2E-F). Cisd2 expression was decreased in these tissue-specific knockout mouse models (Supplementary Fig. 2G-I). Interestingly, the Cisd2 neuron-specific knockout mice had sponge structures in the brain (Supplementary Fig. 2J). A separate experiment measured the hearing preservation rate in the conventional and conditional Cisd2 knockout mouse models undergoing serial ABR and DPOAE testing. The hearing function of Cisd2 F/F mice was similar to that of WT mice as a control group (Fig. 4A). Severe hearing impairment was detected in the Cisd2 cochlea- and neuron-specific KO mouse models (coKO and neKO) as the defect examined in the Cisd2 conventional KO mice (conKO) (Fig. 4A-B). Cisd2 deletion in the cochlea led to a change in F-actin distribution (phalloidin staining) in outer hair cells and decreases in ribbon synapses (C-terminal binding protein 2, CtBP2-positive) in outer and inner hair cells (Fig. 4C). The neurotransmitter release involved in ribbon synapses is critical for cochlear hair cell functions. Interestingly, Cisd2 could interact with Snapin, a part of the SNARE complex for vesicle docking, fusion and release of neurotransmitters in hair cells (Fig. 4D). In the Cisd2 KO mouse models, Snapin expression was compensated for in most parts of the cochlea (Supplementary Fig. 3A-C). Therefore, Cisd2 deletion could induce a decline in synaptic functions, resulting in hearing deterioration and loss in mice.

**Figure 5.**
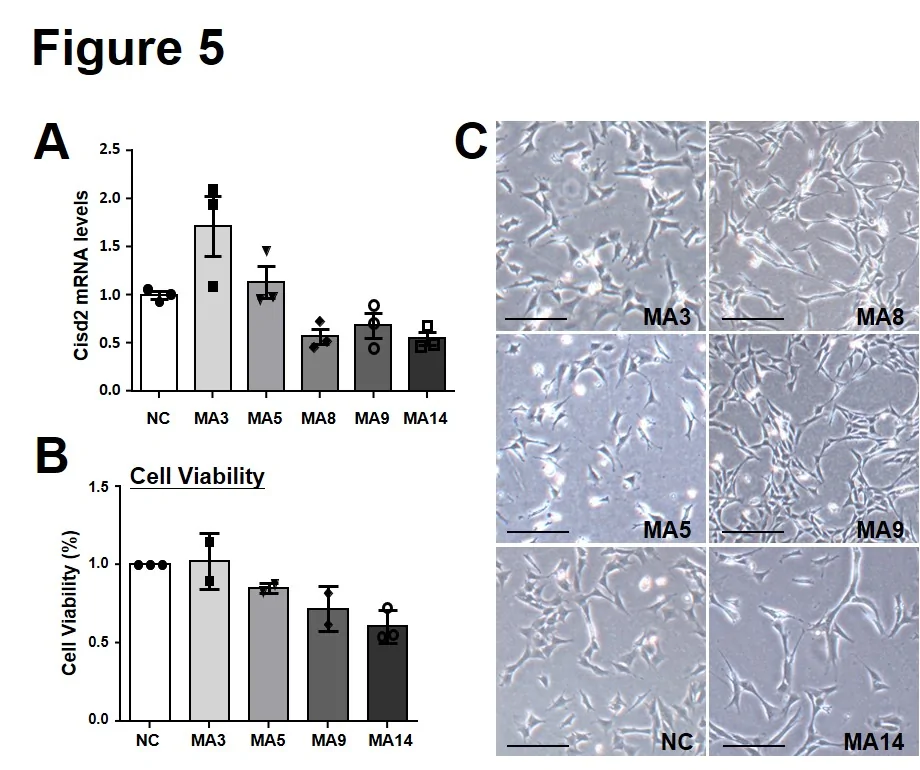
**Characterization of the HEI-OC1 cell line with Cisd2 knockdown**. (**A**) Cisd2 expression levels were detected in HEI-OC1 cell lines transfected with Csid2 shRNA. N = 3 per group. The results are presented as the mean ± SEM. (**B**) Cell viability of Cisd2-knockdown HEI-OC1 cell lines (5 x 10^5^ cells) was measured after 24 hours using the MTT assay. N = 3 per group. The results are presented as the mean ± SD. (**C**) Cell morphology was observed in Cisd2-knockdown HEI-OC1 cell lines. (**A**)-(B), three individual tests were in each clone. NC, control group; MA3, MA4, MA5, MA8, MA9 and MA14 are different clones with Cisd2 knockdown. Scale bar, 0.1 mm.

### The critical mechanisms involved in inducing cell death in Cisd2 KO cochlea

We aimed to investigate the molecular mechanisms that are affected by Cisd2 deletion in mice. We generated an auditory cell line (HEI-OC1) with Cisd2 knockdown (Fig. 5A). Cisd2 knockdown led to a decrease in cell viability and a change in cell morphology (Fig. 5B-C). Lower expression levels of Cisd2 (MA8, MA9, MA14) could not be subcultured for more than three passages. Therefore, the organ of Corti cells cannot be healthy and survive with low Cisd2 levels.

**Figure 6.**
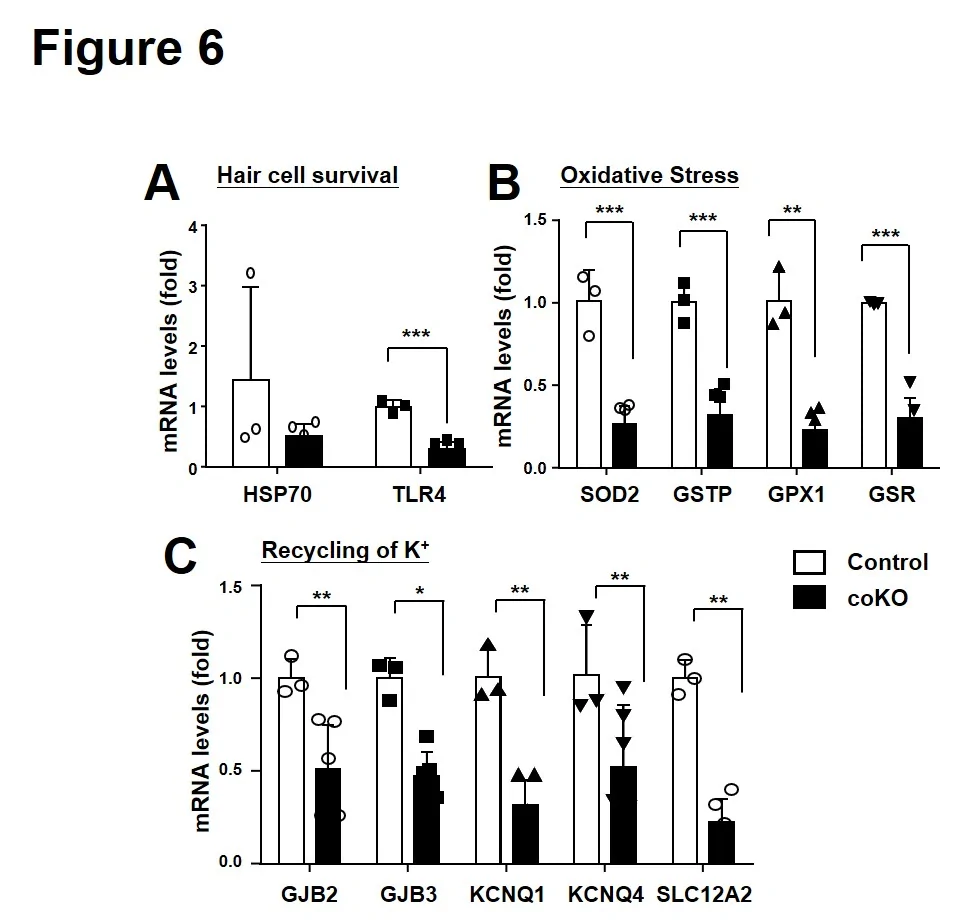
**The expression levels of genes in the cochlea involved in modulating hearing functions**. (**A**) The mRNA levels of the genes for maintaining hair cell survival. (**B**) The mRNA levels of the genes regulating oxidative stress. (**C**) The mRNA levels of the genes regulating the recycling of potassium. (**A**)-(C) Mouse age, 3 months old. Control group n=3 and coKO group n=6. The results are presented as the mean ± SD. *p<0.05; **p<0.01; ***p<0.001.

**Figure 7.**
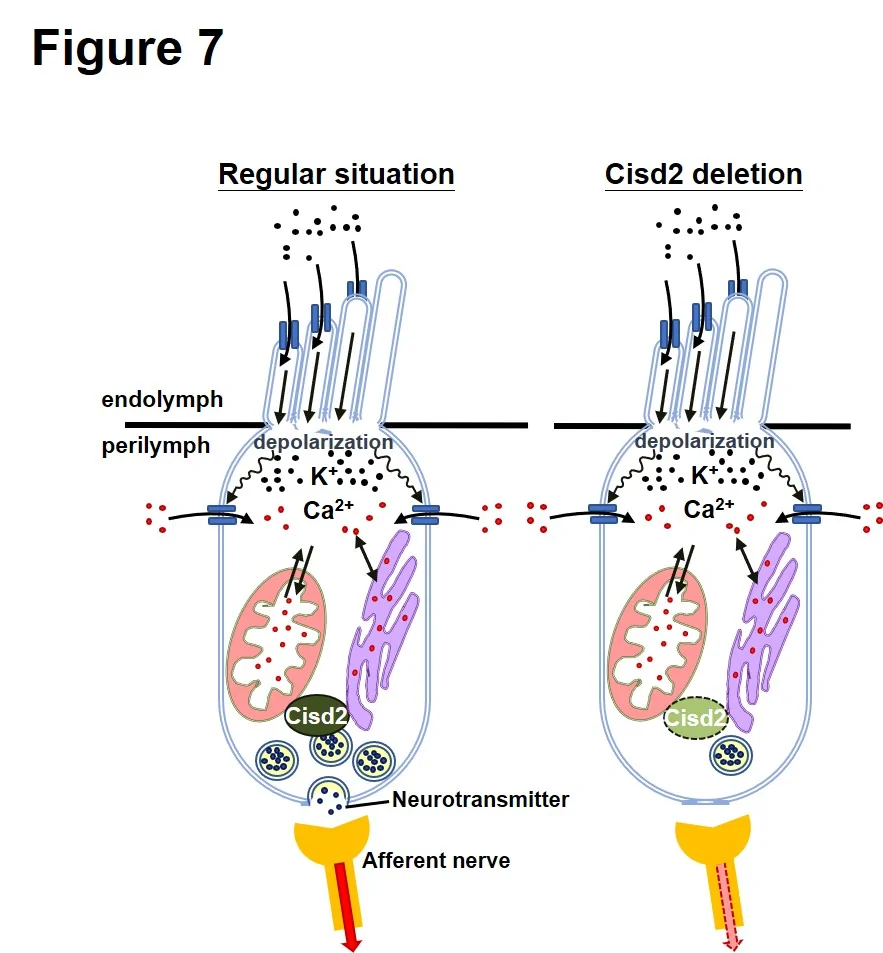
**Cisd2 regulates calcium homeostasis in hair cells**. Sound-induced vibrations convert to the deflection of the stereocilia that induces ion channel opening. Increased intercellular K^+^ induces membrane depolarization that causes more Ca^2+^ to enter the cytoplasm. Neurotransmitter release in hair cells is K^+^-stimulated and Ca^2+^ dependent. However, (1) Cisd2 deficiency leads to the destruction of intercellular ion homeostasis. (2) Cisd2 deficiency reduces the releasable pool of vesicles.

Surrounded supporting cells can modulate the survival of hair cells through paracrine signals, such as the Hsp70-TLR4 interaction [33]. Genetic variations in Hsp70 are highly related to susceptibility to hearing loss [34]. Overexpression of Hsp70 in surrounding supporting cells efficiently inhibited hair cell death; therefore, the heat shock response promotes hair cell survival against stress [35]. Both Hsp70 and TLR4 were downregulated in the cochlea of the Cisd2 KO mouse model (Fig. 6A). The unbalanced redox homeostasis combined with diminished antioxidant defense and cumulative mitochondrial dysfunction seems to be the major mechanism underlying the loss of hair cells [36, 37]. In addition, accumulative oxidative stress could induce premature senescence of auditory cells. In the cochlea of the Cisd2 KO mice, several antioxidant proteins were downregulated, including SOD2, GSTP, GPX1 and GSR (Fig. 6B). Defects in any one of the potassium (K^+^) transporters result in endocochlear potential (EP) decline and hearing loss. Potassium homeostasis is strongly related to cell survival or death. Several susceptibility genes for hearing loss are involved in potassium recycling, such as connexin genes (GJB2, GJB3), potassium channels or channel subunits (KCNQ1, KCNQ4) and Na^+^/2Cl^-^/K^+^ cotransporter (SLC12A2) [38]. These proteins are involved in K^+^ recycling to avoid K^+^ toxicity and maintain hair cell function [39]. In the cochlea of Cisd2 coKO mice, these gene expression levels were significantly lower than those in the cochlea of control mice (Fig. 6C). Consequently, cell death in the cochlea was caused by loss of Cisd2, which could be a critical reason for hearing defects (Fig. 7). As our data showed, Cisd2 neKO mice had similar phenotypes, hearing impairment and compensated Snapin expression, to Cisd2 coKO mice. In this model, neuronal defects may indirectly induce cell damage and the death of hair cells in the cochlea which needs further exploration in the future.

## DISCUSSION

The World Health Organization (2018) reported that one-third of adults over 65 years old experience ARHL. This disease impacts their quality of life and is highly related to isolation, age-related cognitive deficits, and neurological disorders. The primary mechanisms underlying hearing loss are associated with oxidative stress, mitochondrial dysfunction, and aging. Many biomolecular mechanisms are involved in the pathogenesis of ARHL, such as ROS generation, membrane transport, cochlear synaptopathy, hormones and small RNA regulation; therefore, genetic factors are expected to be risk factors of ARHL. Generally, genetic defects and environmental factors work together to induce ARHL, not only one issue, otherwise a genetic defect may cause a serial of stress or damage which can induce and exacerbate ARHL. Under various types of stress, such as aging stress or environmental damage, decreased antioxidant activities and accumulated ROS trigger mitochondrial dysfunction and affect cellular functions [40-42]. An increase in the ABR threshold is observed with age. In addition, active oxygen radicals oxidize and attack macromolecules, such as DNA, protein and lipids, resulting in cell damage and death. The balance between antioxidant defenses and oxidants is critical for mitochondrial function and cell survival. Mitochondria are the major component for energy supplementation and are important for highly energy-dependent cells, such as hair cells and SGNs in the inner ear. Mitochondrial DNA (mtDNA), unlike nuclear DNA with histone protection, is vulnerable and attackable by oxygen radicals. mtDNA mutation and redox imbalance induce cochlear senescence during the aging process. Several nuclear-encoded mitochondrial proteins play a key role in protecting mitochondrial components against ROS damage, such as IDH2, GSR and TXNRD2 [43]. The intrinsic pathway for apoptosis is triggered by the impaired integrity of the outer membrane of mitochondria, which is observed as the key reason for outer hair cell death in aged mice [44]. Mitochondrial biogenesis and selective degradation of mitochondria (mitophagy) are critical processes for mitochondrial turnover. However, the function of autophagy/mitophagy in removing damaged mitochondria is age dependent; accumulated ROS and damaged organelles cause disruption of normal cell functions [45]. Taken together, under aging stress, hearing function is exhausted by mtDNA mutation, defects in oxidative phosphorylation, impairment of antioxidant functions and further increased ROS accumulation [46].

Hair cells are responsible for electromechanical transduction, and the decline of both OHC and IHC are the most prevalent genesis of ARHL [47]. The efferent medial olivocochlear synapses regulate the activity of OHC via making a contact. Medial olivocochlear efferent could ameliorate age-related alteration in OHC [48]. Ribbon synapses are the first afferent neuronal connections between inner hair cells and the peripheral end of SGNs in the auditory nervous system. Interestingly, OHC can transiently form synaptic ribbons when the fibers contacting them, while ribbon and ribbon-free clusters of synaptic vesicles are located at the base of mature-form OHC [49, 50]. A decrease in cortical synaptic density and an increase in synaptic size have been documented in the aging central nervous system in humans and laboratory animals [51, 52]. Synaptic alteration was suggested as a critical, perhaps a primary, issue associated with cognitive decline and senescence [53]; actually, loss of ribbon synapses contributes to reduced hearing susceptibility. Additionally, synaptic reorganization was documented in the pathogenetic mechanisms of ARHL [54].

ROS-mediated modifications impair the activity of ion channels and the expression of their protein components, and accumulated oxidative stress disrupts redox homeostasis in the inner ear [37, 55, 56]. Several ion transporter channels, namely, the NKCC, KCNQ and Kr channels, play critical roles in inner ear function [57]; therefore, these channels are candidates for therapeutic targets to prevent hearing decline or recover hearing functions [58]. KCNQ4, critical for OHC function, and KCNQ1, expressed in marginal cells, are essential components for potassium secretion and circulation. Decreased levels of these two proteins contribute to the progression of age-related hearing loss [59].

Cisd2 is a critical gene involved in maintaining mitochondrial functions and integrity [17]. In the human cases in our study, the decrease in CISD2 was obvious in aged and hearing-deficient individuals. C57BL/6 mice have been documented as a model with ARHL at the aged stage [60]. According to data from genetically modified mouse models, Cisd2 deletion accelerated the disease progression of ARHL in C57BL/6 mice. The main causes of ARHL, loss of cochlear hair cells, SGNs and ribbon synapses, were exacerbated in Cisd2 knockout mouse models. Both hair cells and SGNs are susceptible to injury induced by direct mechanical and mitochondrial oxidation over time. Loss of Cisd2 exacerbated mitochondrial defects, upregulated oxidative stress and disrupted potassium homeostasis, which contributed to decreased synaptic vesicles and hair cell death. For animal studies, we used inbred animals. Using non-inbred animals in future studies will increase genetic variability, thereby enhancing the credibility of the results for human applications. Human participants were recruited locally from Taiwan in a single-center study. In the future, conducting multi-center studies across different ethnic groups will further enhance the credibility and generalizability of the findings. The induction and pathogenesis of ARHL may be modulated by various factors, intrinsic and extrinsic; CISD2 could be one of the critical factors whose expression level was decreased in aged or ARHL human individuals. Therefore, reduced expression of CISD2 may be one predicted target to monitor ARHL. Additionally, this study provides a novel strategy potential to attenuate the pathogenesis of ARHL by elevating CISD2 expression levels for human patients.

## Supplementary Materials

The Supplementary data can be found online at: www.aginganddisease.org/EN/10.14336/AD.2024.1036.
