# Supplementary material for: Loss of Cisd2 Exacerbates the Progression of Age-Related Hearing Loss
Source: Aging Dis. 2024 Aug 24;16(4):2468–82. doi: 10.14336/AD.2024.1036 (PMC12221414; doi:10.14336/AD.2024.1036)
Supplement: Supplementary file 1 — The Supplementary data can be found online at: www.aginganddisease.org/EN/10.14336/AD.2024.1036. [file AD-16-4-2468-s.pdf]

SUPPLEMENTARY DATA

Loss of Cisd2 Exacerbates the Progression of Age-Related Hearing Loss

Hang-Kang Chen, Yen-Hsin Wang, Cing-Syuan Lei, Yu-Ru Guo, Ming-Chi Tang, Ting-Fen Tsai, Yi-Fan Chen, Chih-Hung Wang

© 2024. Chen HK et al. Published online at http://www.aginganddisease.org/EN/10.14336/AD.2024.1036

SUPPLEMENTARY DATA

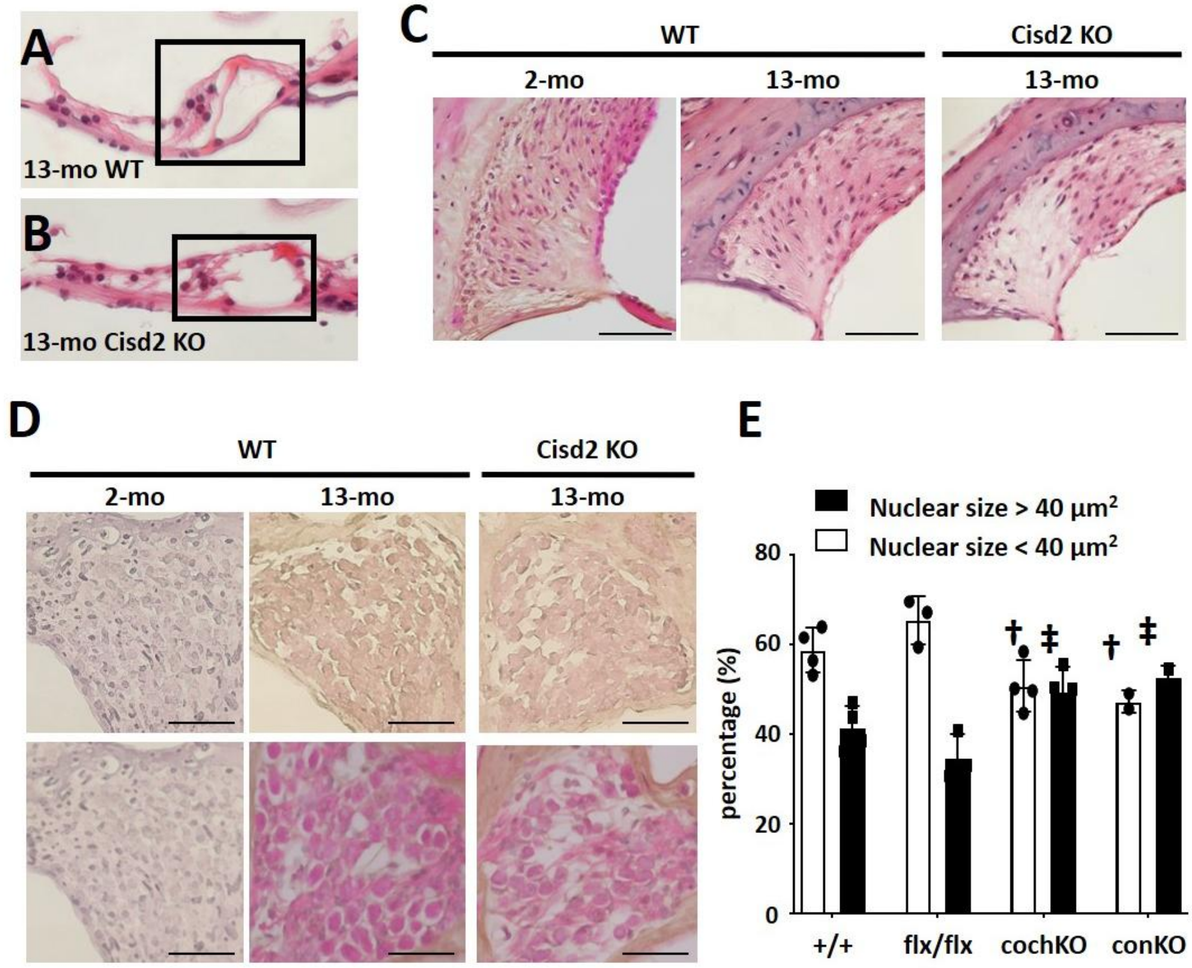

© 2024. Chen HK et al. Published online at http://www.aginganddisease.org/EN/10.14336/AD.2024.1036

SUPPLEMENTARY DATA

Supplemental Figure 1_Continued

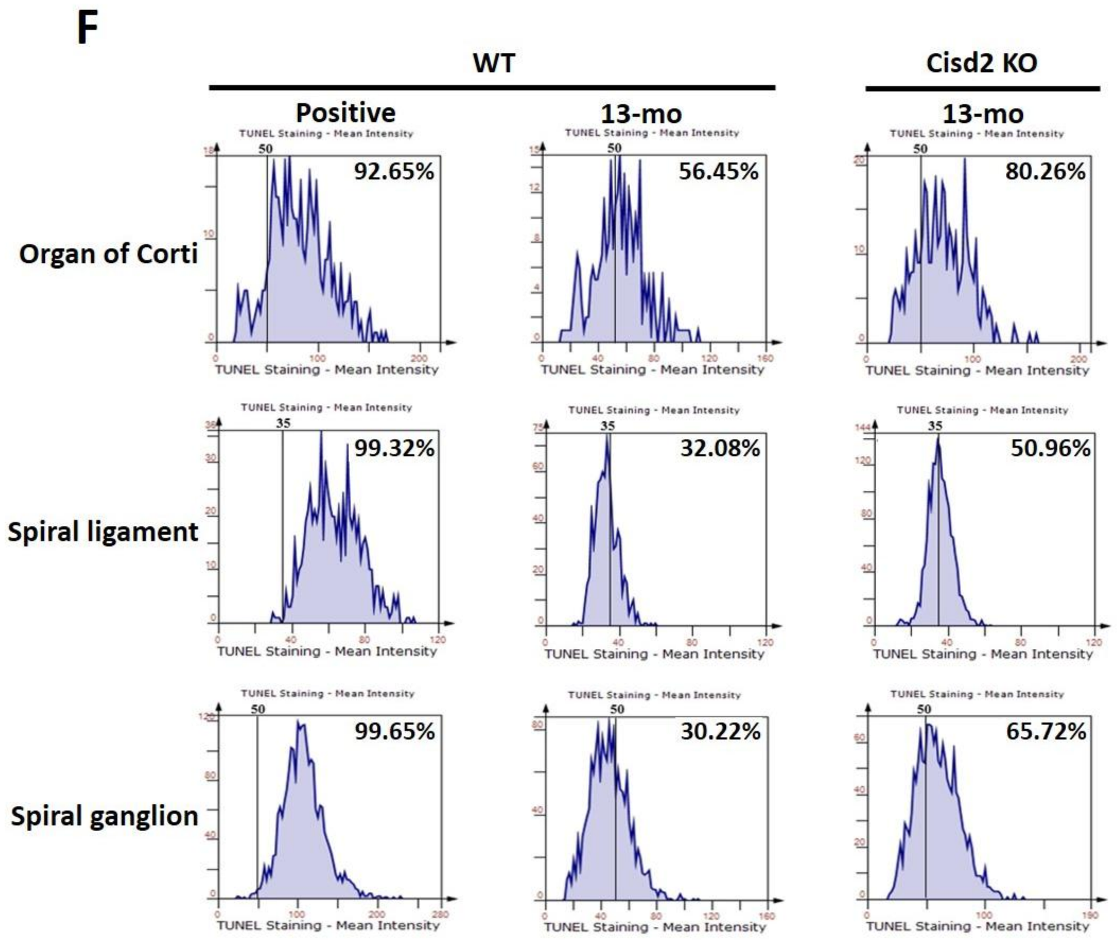

Supplementary Figure 1. Cell death in cochlea with Cisd2 deletion. (A) The cross section of cochlea obtained from 13-month-old WT mice. **(B)** The cross section of cochlea was obtained from the Cisd2 KO mice at 13 months old. **(C)** The spiral ligament of WT mice was wide-spreadly degenerated with advancing age. At 13 months old, the spiral ligament of Cisd2 KO mice was more severe in loss of type IV fibrocytes**. (D)** Cell loss was observed in spiral ganglion of Cisd2 KO mice. **(E)** Quantitative analysis of cochlear ganglions was performed using HistoQuest system. WT mice n= 4; Cisd2 flxf/flx mice n=3; cochlea-specific knockout (coKO) mice n=4 and Cisd2 conventional KO (conKO) mice n=2. Data are presented as mean ± SD. †Significantly differs from the Cisd2 flx/flx group (black bar, nuclear size > 40 μm2), p< 0.05. ‡Significantly differs from the Cisd2 flx/flx group (white bar, nuclear size < 40 μm2), p< 0.05. **(F)** The quantification of apoptosis in the WT and Cisd2 KO mice cochlea by TUNEL staining in Figure 3A. Positive controls were the tissue slides treated with DNase I 100 U/mL for 15 mins. Scale bar, 50μm.

© 2024. Chen HK et al. Published online at http://www.aginganddisease.org/EN/10.14336/AD.2024.1036

SUPPLEMENTARY DATA

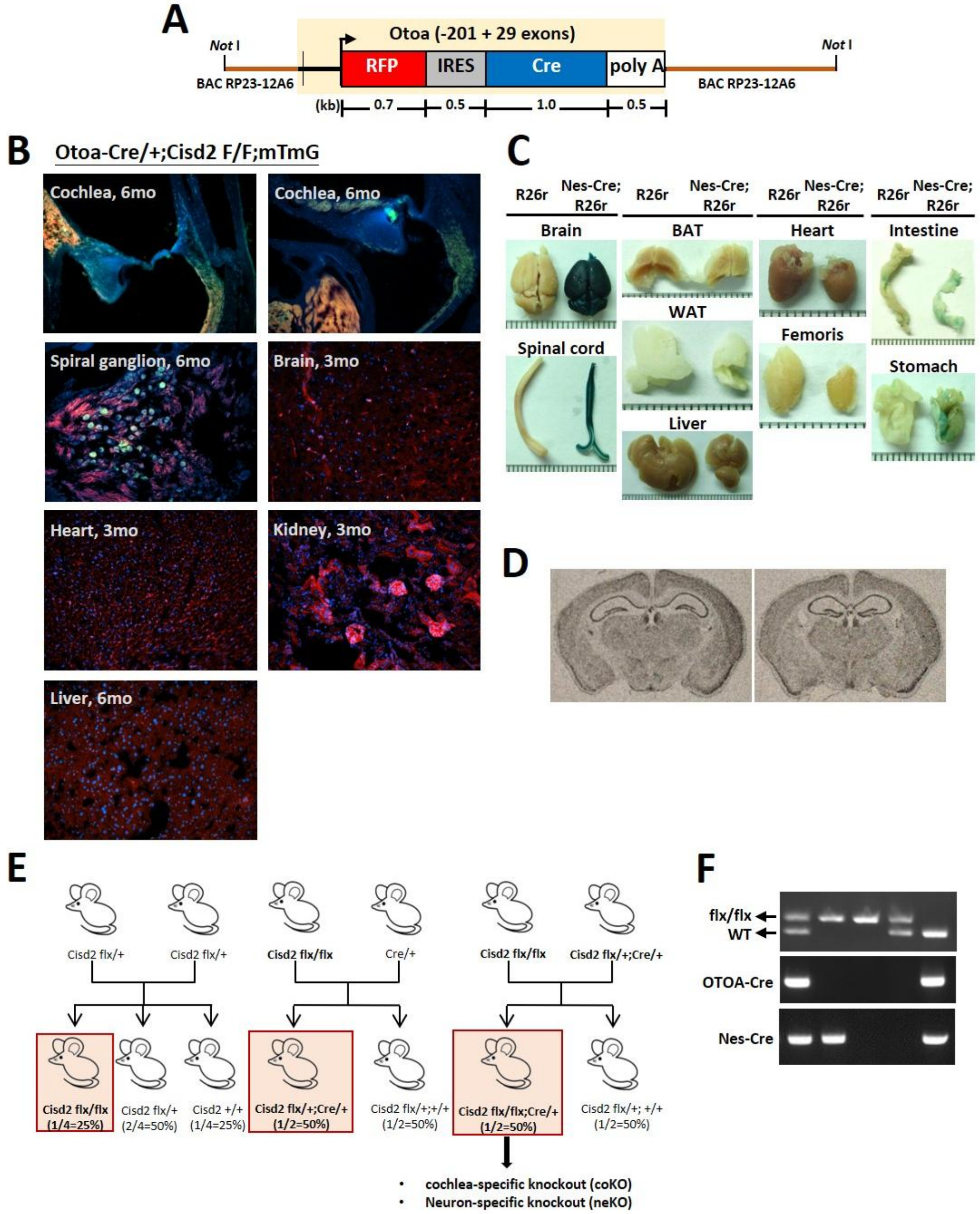

© 2024. Chen HK et al. Published online at http://www.aginganddisease.org/EN/10.14336/AD.2024.1036

SUPPLEMENTARY DATA

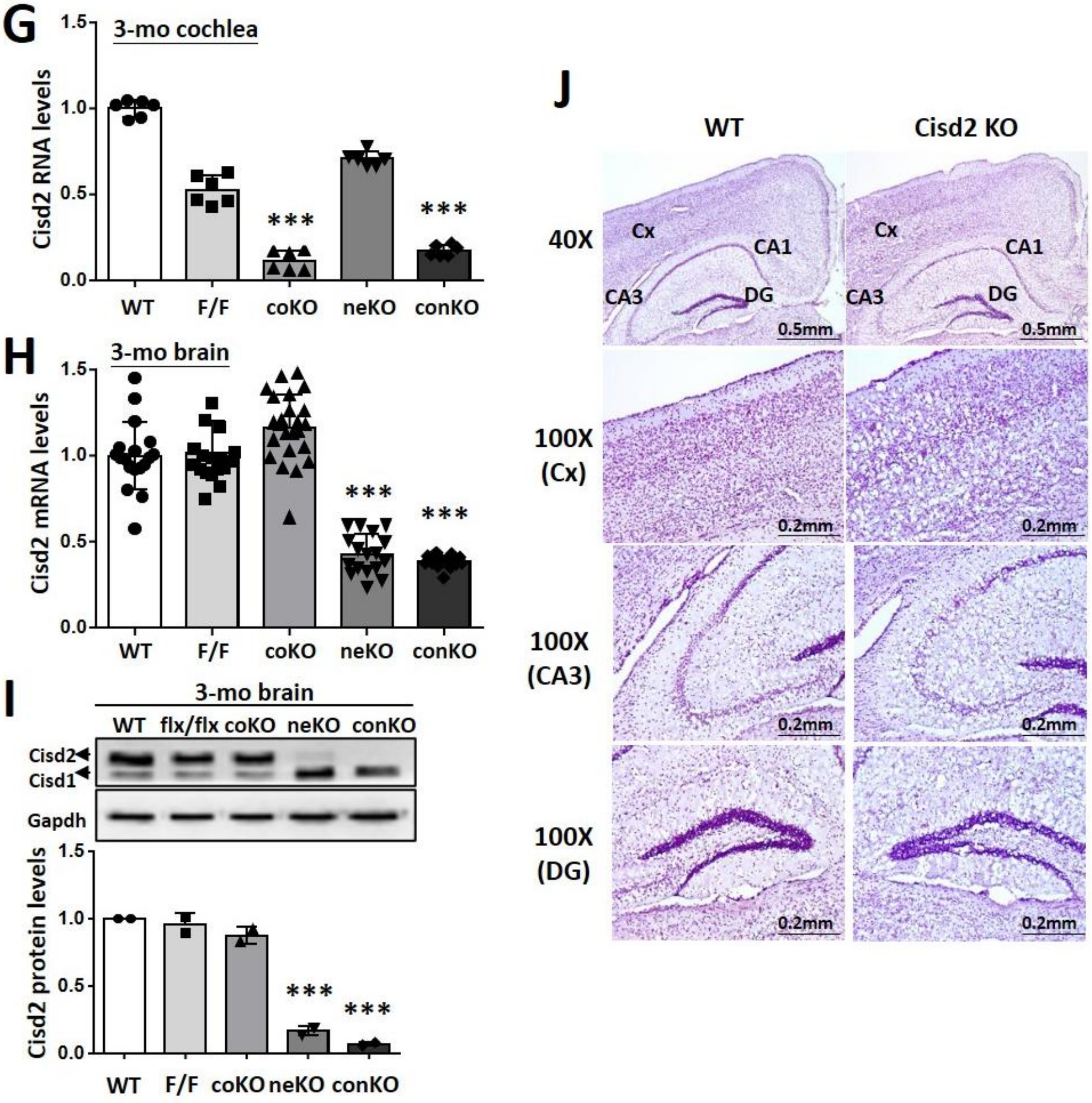

Supplementary Figure 2. Generation and analysis of the Cisd2 inner ear- and neuron-specific KO mice. (A) The construction of Otoa-Cre transgenic mouse model. **(B)** The histological analysis of Otoa- Cre/+;Cisd2 F/F;mTmG mice to detect the expression site of Cre recombinase. Orange, tdTomato; green, eGFP; blue, DAPI. **(C)** β-galactosidase activity of various tissues from Nestin (Nes)-Cre;ROSA26R mice. Nestin-Cre was majorly expressed in the nervous system. No obvious expression was observed in other tissues. **(D)** *in situ* hybridization of Cisd2 in brain section. **(E)** The breeding strategy for generation of Rrm2b tissue-specific knockout mouse models. **(F)** The genotyping for Rrm2b tissue- specific knockout mouse models. **(G)** Decreased Cisd2 mRNA expression were observed in cochlea of Otoa-Cre/+;Cisd2 f/f and Cisd2 conventional knockout mouse models at 3 months of age. Mouse number in each group is 6. **(H)** Decreased Cisd2 mRNA expression were observed in brains of Nes- Cre/+;Cisd2 f/f and conventional knockout mouse models at 3 months of age. WT mice n= 18; Cisd2 flxf/flx mice n=18; cochlea-specific knockout (coKO) mice n=24; neuron-specific knockout (neKO) mice n= 17 and Cisd2 conventional KO (conKO) mice n=15. **(I)** Decreased Cisd2 protein expression were observed in brains of Nes-Cre/+;Cisd2 f/f and conventional knockout mouse models at 3 months of age. N=2 mice per group. **(J)** Cresyl violet staining in the Cisd2 KO and WT brain. Cx, cortex in brain.

© 2024. Chen HK et al. Published online at http://www.aginganddisease.org/EN/10.14336/AD.2024.1036

SUPPLEMENTARY DATA

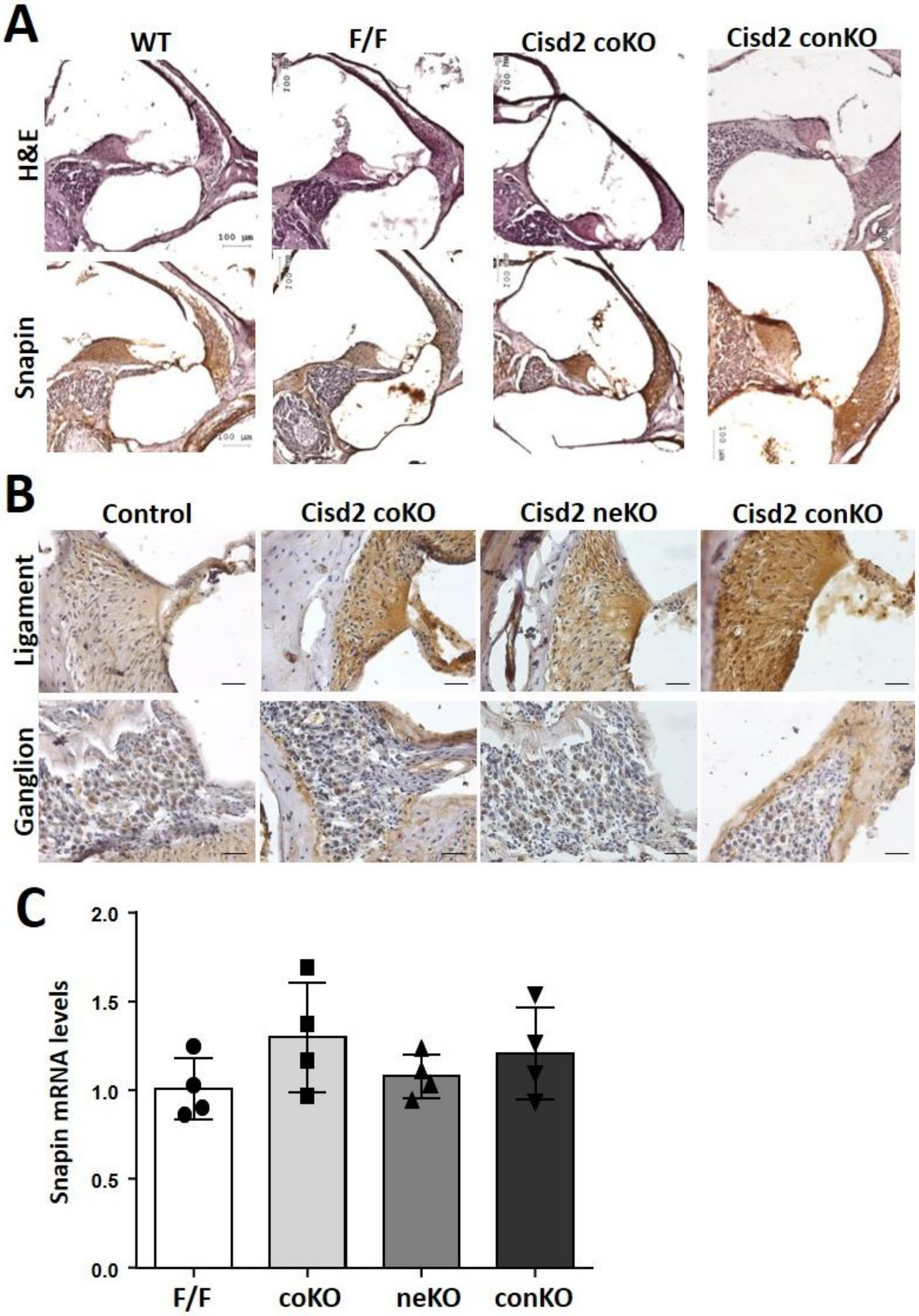

Supplementary Figure 3. The expression levels of Snapin in cochlea when Cisd2 deletion. (A) H&E staining was applied in morphological assessment of cochleae and IHC staining was used to detect Snapin protein expression in cochlea tissues. **(B)** Snapin expression levels were detected in ligaments and ganglions of Cisd2 deleted mouse models and controls. Scale bar, 25μm. **(C)** The mRNA expression levels of Snapin in cochlea of mice. Cisd2 flxf/flx mice n=4; cochlea-specific knockout (coKO) mice n=4; neuron-specific knockout (neKO) mice n=4 and Cisd2 conventional KO (conKO) mice n=4.

© 2024. Chen HK et al. Published online at http://www.aginganddisease.org/EN/10.14336/AD.2024.1036
